# Economic Outcomes and Incidence of Postsurgical Hypotension With Liposomal Bupivacaine vs Epidural Analgesia in Abdominal Surgeries

**DOI:** 10.36469/001c.37739

**Published:** 2022-09-14

**Authors:** Margaret Holtz, Nick Liao, Jennifer H. Lin, Carl V. Asche

**Affiliations:** 1 Georgia Anesthesiologists LLC, Marietta, Georgia; 2 Pacira Biosciences, Inc, Parsippany, New Jersey; 3 Department of Internal Medicine University of Illinois College of Medicine, Peoria, Illinois

**Keywords:** epidural analgesia, liposomal bupivacaine, length of stay, hospital costs, hypotension, abdominal surgery, colorectal surgery

## Abstract

**Background:** Epidural analgesia can be associated with high costs and postsurgical risks such as hypotension, despite its widespread use and value in providing opioid-sparing pain management. We tested the hypothesis that liposomal bupivacaine (LB) might be a reliable alternative to epidural analgesia in this real-world study.

**Objectives:** To compare economic outcomes and hypotension incidence associated with use of LB and epidural analgesia for abdominal surgery.

**Methods:** This retrospective analysis identified records of adults who underwent abdominal surgeries between January 2016 and September 2019 with either LB administration or traditional epidural analgesia using the Premier Healthcare Database. Economic outcomes included length of stay, hospital costs, rates of discharge to home, and 30-day hospital readmissions. Secondary outcomes included incidence of postsurgical hypotension and vasopressor use. Subgroup analyses were stratified by surgical procedure (colorectal, abdominal) and approach (endoscopic, open). A generalized linear model adjusted for patient and hospital characteristics was used for all comparisons.

**Results:** A total of 5799 surgical records (LB, n=4820; epidural analgesia, n=979) were included. Compared with cases where LB was administered, cases of epidural analgesia use were associated with a 1.6-day increase in length of stay (adjusted rate ratio [95% confidence interval (CI), 1.2 [1.2-1.3]]; *P*<.0001) and $6304 greater hospital costs (adjusted rate ratio [95% CI], 1.2 [1.2-1.3]]; *P*<.0001). Cost differences were largely driven by room-and-board fees. Epidural analgesia was associated with reduced rates of discharge to home (*P*<.0001) and increased 30-day readmission rates (*P*=.0073) compared with LB. Epidural analgesia was also associated with increased rates of postsurgical hypotension (30% vs 11%; adjusted odds ratio [95% CI], 2.8 [2.3-3.4]; *P*<.0001) and vasopressor use (22% vs 7%; adjusted odds ratio [95% CI], 3.1 [2.5-4.0]; *P*<.0001) compared with LB. Subgroup analyses by surgical procedure and approach were generally consistent with overall comparisons.

**Discussion:** Our results are consistent with previous studies that demonstrated epidural analgesia can be associated with higher utilization of healthcare resources and complications compared with LB.

**Conclusions:** Compared with epidural analgesia, LB was associated with economic benefits and reduced incidence of postsurgical hypotension and vasopressor use.

## INTRODUCTION

Epidural analgesia is commonly implemented following abdominal surgeries to reduce complications and provide opioid-sparing pain management in enhanced recovery after surgery protocols.^1–3^ Despite widespread use, epidural analgesia can be associated with high costs. For example, in a retrospective database analysis of approximately 192 000 patients who underwent laparoscopic colorectal procedures in the United States between 2002 and 2010, epidural analgesia was associated with an increase in hospital costs of $3733 compared with procedures that did not use epidural analgesia.^4^ Similarly, a European study of patients undergoing major abdominal surgery showed that although epidural use provided more effective pain relief when compared with intravenous (IV) patient-controlled analgesia (PCA), the incremental cost-effectiveness ratio relative to IV PCA was associated with an additional €5653 (~$6713 US dollars [in 2015 costs]) per each pain-free day.^5^ It has been suggested that epidural analgesia may increase hospital costs, given the need for intensive patient monitoring; however, there is a lack of recent data pertaining to its cost-effectiveness.^6^ Additionally, epidural analgesia is associated with clinical risks, as patients given epidural analgesia are prone to nausea, vomiting, motor weakness, urinary retention, pruritus, and dizziness, along with rare but serious complications such as epidural abscess and hematoma.^7–9^ Epidural analgesia has also long been associated with postsurgical hypotension, which has been reported to occur in approximately 33% of patients.^10^ At least 2 meta-analyses have demonstrated increased risk of hypotension with epidural analgesia compared with transversus abdominis plane (TAP) block in abdominal surgery.^1,11^ However, there are limited data comparing healthcare resource utilization, costs, and postsurgical hypotension rates between epidural analgesia and alternative regional analgesic methods in the same study population.

Regional analgesic alternatives to epidural analgesia include perineural techniques, intrathecal morphine, infiltrative regional anesthesia, intravenous analgesia, and fascial plane blocks.^6^ Use of opioids is also an option but may be associated with a risk of prolonged opioid use, which has downstream negative consequences.^12^ An alternative is liposomal bupivacaine (LB), a long-acting multivesicular liposome formulation of the local anesthetic bupivacaine that provides prolonged bupivacaine release after infiltration.^13^ Previous data have suggested that LB administered via fascial plane blocks may be a reliable alternative to epidural analgesia. In a retrospective study of 318 patients who underwent major lower abdominal surgery, administration of LB via TAP block was noninferior to epidural analgesia in reducing pain scores after lower abdominal surgery.^14^ Furthermore, in a randomized clinical trial of 179 patients undergoing colorectal surgery, LB TAP block provided equivalent analgesia to epidural analgesia and significantly reduced opioid use and costs.^2^ The use of LB TAP block was also associated with a 0.5-day reduction in length of stay (LOS) compared with epidural analgesia in another randomized trial of approximately 80 patients undergoing colorectal surgery.^15^ However, more research in real-world clinical practice detailing the specific potential economic and clinical benefits of substituting LB fascial plane blocks for continuous epidural analgesia in abdominal and colorectal surgeries is needed.

In the present study, we tested the primary hypothesis whether measures associated with hospital costs and duration of hospitalization were increased in patients given epidural analgesia. Additionally, we tested the secondary hypothesis that postsurgical hypotension was more common in patients given epidural analgesia than in those given peripheral LB.

## METHODS

### Study Population

This retrospective cohort analysis used the Premier Healthcare Database, which contains administrative data since January 2000 from over 1000 US hospitals and represents approximately 25% of US inpatient admissions.^16^ The database contains data on hospital characteristics, patient visit characteristics, specialties of admitting and attending physicians, healthcare payers, and patient information based on standard hospital discharge billing files.^16^ Economic data in the Premier Healthcare Database are based on a comprehensive charge master table comprising items billable to a hospital patient or a health insurance provider. Costs are determined by the accounting systems of each hospital or through the ratios of cost to charges, with charge data supplied by the hospitals to the Premier Healthcare Database. Data from the Premier Healthcare Database have been used in publications of epidemiology and economic research since 2006. This analysis was exempt from institutional review board review requirements because the records are entirely deidentified.^16^

**Figure 1.**
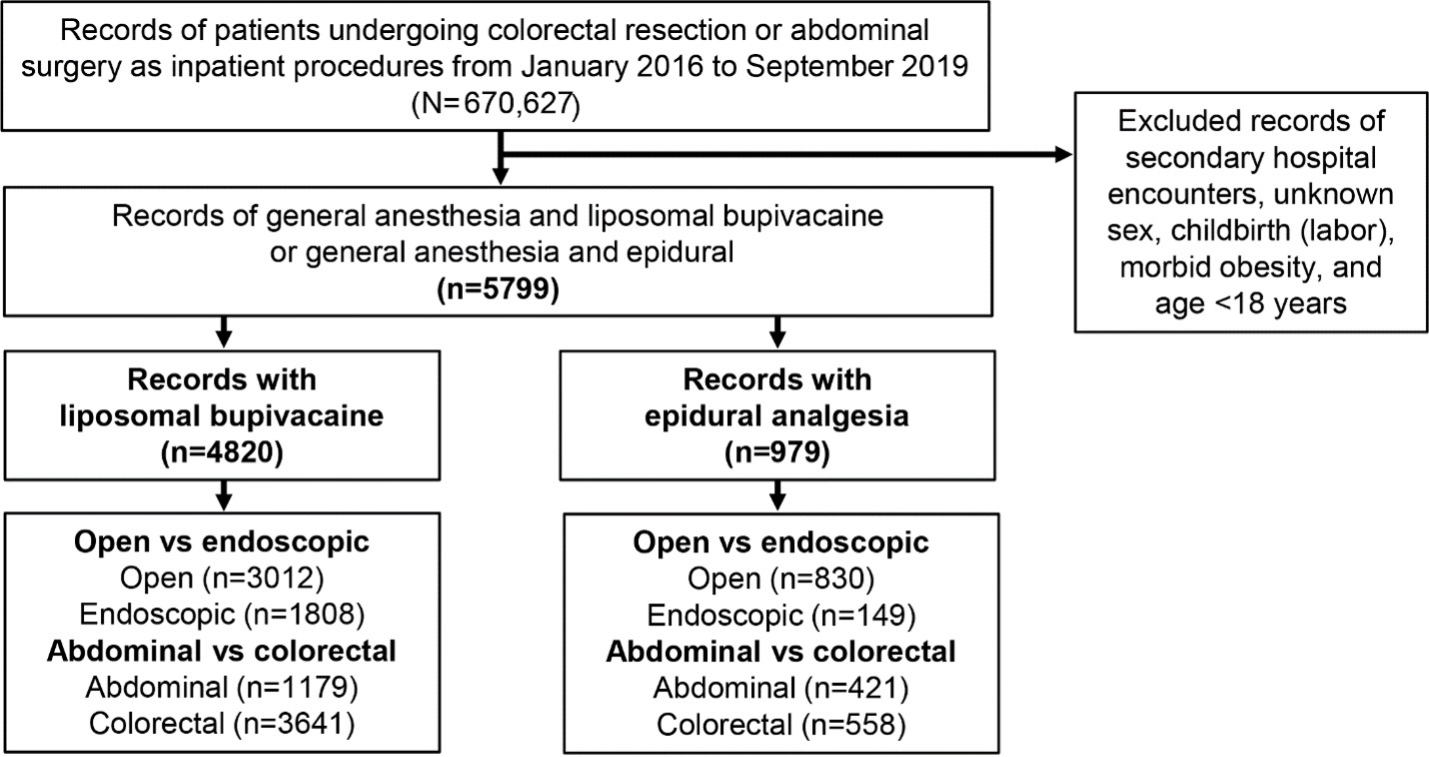
Identification Process for Retrieving Surgical Records Involving Epidural Analgesia vs Liposomal Bupivacaine Records retrieved from the Premier Healthcare Database, with the number of each approach (eg, open vs endoscopic) and surgical procedure (abdominal vs colorectal resection).

Data were analyzed from inpatients who had received abdominal surgical procedures during the most recent years between January 2016 and September 2019 (end of data license). Colorectal resections were separated from other abdominal surgeries for analysis (**Supplementary Table S1**). We identified records of patients receiving general anesthesia by standard charge codes (**Supplementary Table S2**) combined with either epidural analgesia or LB for postsurgical pain management.^17^ Patients given epidural analgesia were identified by epidural charge codes, which may have included epidural administration of fentanyl, hydromorphone, or meperidine (**Supplementary Table S3**); patients given LB were identified by charge codes listed in **Supplemental Table S4**. Exclusion criteria included age under 18 years, obstetrical patients (*International Statistical Classification of Diseases and Related Health Problems, Tenth Edition* [ICD-10] codes O60-O77 and O80-O82), and morbid obesity (ICD-10 codes E66.01 and E66.02; Figure 1).

### Outcomes

The primary outcomes of interest were economic outcomes, including hospital costs (US dollars), LOS (days), discharge directly to home (yes/no), and 30-day readmissions (yes/no). Total hospital costs included hospital services, medical procedures, equipment fees, supplies, drugs, and diagnostic evaluations such as imaging and laboratory tests. All costs have been converted to 2022 US dollar values using the US Bureau of Labor Statistics Consumer Price Index for healthcare costs. The secondary outcome of interest was incidence of hypotension, which was defined by either postsurgical (ie, not present on admission) diagnosis of hypotension (ICD-10 codes I95.0, I95.1, I95.2, I95.3, I95.81, I95.89, and I95.9), or postsurgical use of vasopressors (ie, ≥1 day after surgery), which included ephedrine, epinephrine, mephentermine, norepinephrine, phenylephrine, and vasopressin.

### Statistical Analysis

A generalized linear model (GLM) was used to compare epidural analgesia vs LB for postsurgical pain management on economic outcomes (LOS, total hospital cost, discharge to home, 30-day readmissions) and clinical outcomes (postsurgical hypotension incidence, vasopressor use) separately by surgical procedure (colorectal resections and abdominal surgeries), by approach (open and endoscopic), and in combination. We used a negative binomial distribution and log link function for LOS. For total hospital costs, we fitted our model with a Tweedie distribution, with the assumption that the variance is proportional to a power of the mean, with log link function. We observed an estimated power of close to 2 (1.87 [95% confidence interval (CI),1.85-1.89]), suggesting the Tweedie distribution for total cost was equivalent to a gamma distribution.^18,19^ We also assumed a binomial distribution for the binary outcomes of discharge to home, 30-day readmission, hypotension incidence, and vasopressor use, with logit link function. Selection of logit link for the 4 binary outcomes was deemed appropriate by a Hosmer-Lemeshow goodness-of-fit test (*P* ≥ .11).^20,21^ We used average marginal effects for adjusted estimates of costs for epidural vs LB use. The complete GLM tables for the economic and clinical outcomes are provided in **Supplementary Table S5**.

Rate ratios between epidural analgesia and LB were provided for LOS and total hospital costs, and odds ratios were calculated for discharge to home, 30-day readmissions, hypotension incidence, and vasopressor use. The analytic model was adjusted for potential confounding factors including patient characteristics (age, sex, race, Quan-Charlson comorbidity index score,^22^ surgical year, and insurance type) and hospital characteristics (hospital type [ie, teaching hospital], provider region [Midwest, Northeast, South, West], location [urban, rural], and bed size [000-199, 200-299, 300-399, 400-499, and ≥500]), and for surgical procedure (in the analysis of combined procedures only). These covariates have been selected in our prior health outcome studies of patients undergoing inpatient surgery.^23,24^ Difference in continuous covariates (age, Quan-Charlson Comorbidity Index) between the 2 analgesia groups were tested using the Wilcoxon rank sum test and categorical variables were tested by the χ^2^ test. All statistical analyses were performed using SAS, version 9.4 (SAS Institute, Cary, North Carolina). A *P* value <.05 was considered statistically significant.

## RESULTS

### Patient Records and Characteristics

From January 2016 to September 2019, 670 627 inpatient abdominal surgeries, including colorectal resection surgeries, were identified. Subsequently, records were removed if the surgery was recorded as a secondary hospital encounter; also excluded were surgeries accompanied by unknown sex, childbirth (labor), morbid obesity, and age under 18 years, which yielded a total of 5799 surgical records of either LB (n = 4820) or epidural analgesia (n = 979) administration for postsurgical pain management. The proportion of records involving open vs endoscopic and abdominal vs colorectal surgery is shown in Figure 1.

Baseline patient and hospital characteristics were generally similar between the epidural and LB treatment groups (Table 1). Overall, approximately half the patients were female, the overall mean age of patients was 62 years, and the mean Quan-Charlson Comorbidity Index score was 2.1. Approximately half of surgical records within each treatment group involved patients insured by Medicare.

**Table 1.** Patient and Hospital Characteristics

	**Epidural Analgesia (n=979)**	**Liposomal Bupivacaine (n=4820)**	**Total (N=5799)**	***P* Value**
Age (y), mean (SD)	62 (16)	62 (16)	62 (16)	.4944
Female, n (%)	486 (50)	2626 (54)	3112 (54)	<.0001
Race, n (%)				
White	808 (83)	3877 (80)	4685 (81)	.0056
Other	171 (17)	943 (20)	1114 (19)	
Quan-Charlson Comorbidity Index score, mean (SD)	2.4 (2.8)	2.0 (2.5)	2.1 (2.6)	<.0001
Surgical procedure, n (%)				
Abdomen	421 (43)	1179 (24)	1600 (28)	<.0001
Colorectal	558 (57)	3641 (76)	499 (72)	
Index surgery year, n (%)				
2016	284 (29)	1352 (28)	1636 (28)	<.0001
2017	198 (20)	1474 (31)	1672 (29)	
2018	219 (22)	1081 (22)	1300 (22)	
2019	278 (28)	913 (19)	1191 (21)	
Payer, n (%)				
Commercial	306 (31)	1777 (37)	2083 (36)	<.0005
Medicaid	94 (10)	332 (7)	426 (7)	
Medicare	497 (51)	2277 (47)	2774 (48)	
Other	82 (8)	434 (9)	516 (9)	
Teaching hospital, n (%)				
No	382 (39)	2917 (61)	3299 (57)	<.0001
Yes	597 (61)	1903 (39)	2500 (43)	
Hospital location, n (%)				
Rural	237 (24)	355 (7)	592 (10)	<.0001
Urban	742 (76)	4465 (93)	5207 (90)	
Provider region, n (%)				
Midwest	141 (14)	383 (8)	524 (9)	<.0001
Northeast	149 (15)	87 (18)	1828 (18)	
South	608 (62)	3316 (69)	3924 (68)	
West	81 (8)	242 (5)	323 (6)	
Bed size, n (%)				
0-199	42 (4)	808 (17)	850 (15)	<.0001
200-299	86 (9)	615 (13)	701 (12)	
300-399	114 (12)	988 (21)	1102 (19)	
400-499	93 (10)	202 (4)	295 (5)	
≥500	644 (66)	2207 (46)	2851 (49)	

### Economic Outcomes

**Figure 2.**
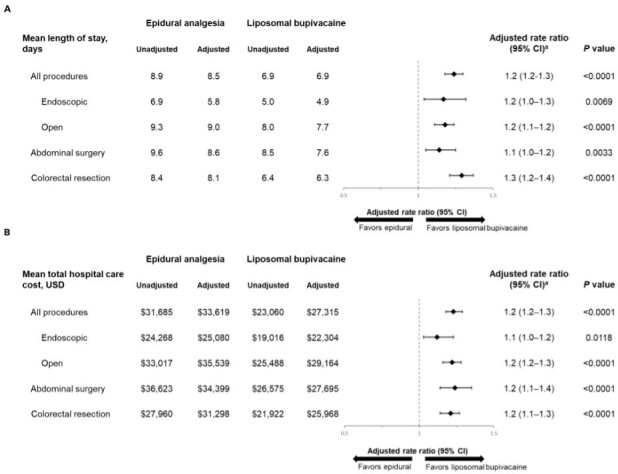
Economic Outcomes: LOS Ratio (**A**) and Cost Ratio (**B**) by Procedure Type and Surgery in Patients Given Epidural Analgesia or LB Model adjusted for age, sex, race, Quan-Charlson Comorbidity Index score, insurance type, hospital type, provider region, location, surgical year, bed size, and surgical procedure (all procedures analysis only). Liposomal bupivacaine was used as the reference in calculation of rate ratios. Abbreviations: CI, confidence interval; LB, liposomal bupivacaine; LOS, length of stay; USD, US dollars. ^a^Values were rounded to 1 decimal place.

Across both surgical procedures, patients given epidural analgesia stayed in the hospital an extra 1.6 days vs those given LB (adjusted LOS: 8.5 vs 6.9 days, respectively). For individual surgical procedures (ie, abdominal vs colorectal), hospital LOS was significantly longer in patients given epidural analgesia compared with those given LB for abdominal surgery (adjusted LOS rate ratio: 1.1; *P* = .0033) and colorectal resection (adjusted LOS ratio: 1.3; *P* < .0001; Figure 2A). Similarly, hospital LOS was significantly longer in patients who received epidural analgesia compared with those given LB for both the endoscopic and open surgery subgroups (adjusted LOS rate ratios: 1.2; *P* < .01 for both).

Total adjusted hospital costs for the 2 surgical procedures combined were $6304 greater for patients given epidural analgesia than for those given LB (*P* <  0001; Figure 2B). Total adjusted hospital costs were also significantly greater in patients given epidural analgesia compared with those given LB in individual surgeries of the abdomen and colorectal resection (*P*  < .0001 for both). When both surgeries together were analyzed by approach (endoscopic vs open), total adjusted hospital costs were $6375 greater for patients who received epidural analgesia compared with those who received LB during an open abdominal or colorectal surgical procedure and $2776 greater in patients who received an endoscopic procedure.

Itemized cost analysis, across surgical procedures and approaches, showed that epidural analgesia was associated with greater medical care costs compared with LB, with significant cost increases related to room and board, anesthesia, blood bank fees, respiratory support, and professional fees (Table 2). Adjusted pharmacy costs were not statistically significant between the 2 groups (adjusted rate ratio [95% CI], 1.00 [0.94-1.07]; *P* = .8840). The overall results remained mostly unchanged when costs were analyzed separately for abdominal surgery and colorectal resection.

**Table 2.** Itemized Hospital Cost Comparisons in Abdominal and Colorectal Resection Surgeries

**Cost Attribute**	**Epidural Analgesia^a^**		**Liposomal Bupivacaine^a^**		**Adjusted Rate Ratio (95% CI)^b^**	***P* Value**
	**Unadjusted**	**Adjusted**	**Unadjusted**	**Adjusted**		
**Overall analysis**						
Medical	25 938	30 399	18 646	24 134	1.26 (1.21-1.32)	<.0001
Room and board	11 661	12 634	7800	8916	1.42 (1.34-1.50)	<.0001
Anesthesia	1046	1128	616	785	1.44 (1.32-1.57)	<.0001
Blood bank	704	503	265	223	2.26 (1.78-2.86)	<.0001
Respiratory support	747	991	298	410	2.42 (1.59-3.67)	<.0001
Professional fee	940	700	5	4	197.96 (145.52-269.30)	<.0001
Pharmacy	3291	3108	2648	3093	1.00 (0.94-1.07)	0.884
**Analysis by surgical procedure**						
Abdominal surgeries						
Medical	29 847	30 873	21 240	24 096	1.28 (1.18-1.39)	<.0001
Room and board	13 765	13 047	10 215	9842	1.33 (1.19-1.47)	<.0001
Anesthesia	1121	899	598	783	1.15 (0.96-1.37)	0.1284
Blood bank	970	560	435	233	2.40 (1.62-3.59)	<.0001
Respiratory support	1009	760	502	410	1.85 (1.18-2.93)	0.008
Professional fee	839	151	5	0	394.69 (255.62-609.41)	<.0001
Pharmacy	3949	3325	3324	3531	0.94 (0.83-1.07)	0.3414
Colorectal resections						
Medical	22 988	28 510	17 806	23 204	1.23 (1.17-1.29)	<.0001
Room and board	10 074	11 509	7018	7907	1.46 (1.35-1.57)	<.0001
Anesthesia	989	1245	622	808	1.54 (1.39-1.71)	<.0001
Blood bank	502	378	210	190	1.99 (1.46-2.71)	<.0001
Respiratory support	549	871	233	311	2.81 (1.41-5.57)	0.0032
Professional fee	1017	1366	5	5	296.14 (191.71-457.45)	<.0001
Pharmacy	2794	2734	2430	2705	1.01 (0.94-1.09)	0.7768

Abbreviation: CI, confidence interval.

^a^Amounts reported in USD.

^b^Model was adjusted for age, sex, race, Quan-Charlson Comorbidity Index score, teaching hospital, provider region, location, index surgery year, surgery type, bed size, and payor.

**Figure 3.**
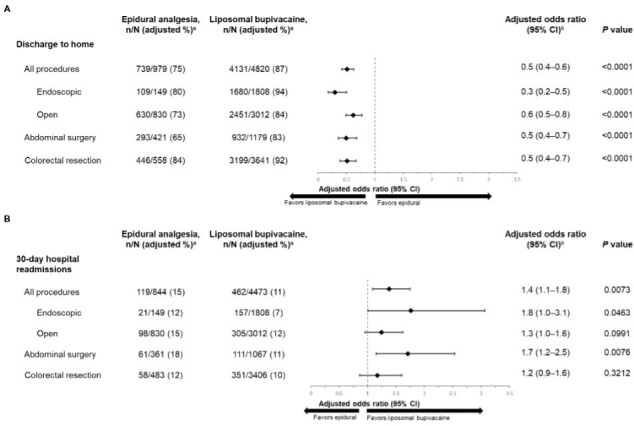
Economic Outcomes: Discharge to Home (**A**) and 30-Day Hospital Readmissions (**B**) by Procedure Type and Surgery in Patients Given Epidural Analgesia or LB Model adjusted for age, sex, race, Quan-Charlson Comorbidity Index score, insurance type, hospital type, provider region, location, surgical year, bed size, and surgical procedure (for procedures analysis only). Liposomal bupivacaine was used as the reference in odds ratio calculations. Abbreviations: CI, confidence interval; LB, liposomal bupivacaine. ^a^Percentages were rounded to the nearest whole number. ^b^Values were rounded to 1 decimal place.

Across both surgical procedures, patients who received epidural analgesia were significantly less likely to be discharged directly to home than patients who received LB (adjusted rate: 75% vs 87%; *P* < .0001; Figure 3A). Similar results were also observed when stratified by surgical procedures (abdomen or colorectal resection) and approach (endoscopic or open) (Figure 3A).

The use of epidural analgesia was associated with an increased rate of 30-day hospital readmissions compared with LB when both abdominal and colorectal resection surgeries were combined (adjusted rate: 15% vs 11%; *P* = .0073; Figure 3B). When subgroup analyses were performed by surgical approach and procedure, epidural analgesia was associated with significantly greater rates of 30-day hospital readmission than LB for endoscopic approach (*P* = .0463) and abdominal surgery (*P* = .0076) but not open approach (*P* = .0991) or colorectal resection (*P* = .3212).

### Postsurgical Hypotension and Vasopressor Use

**Figure 4.**
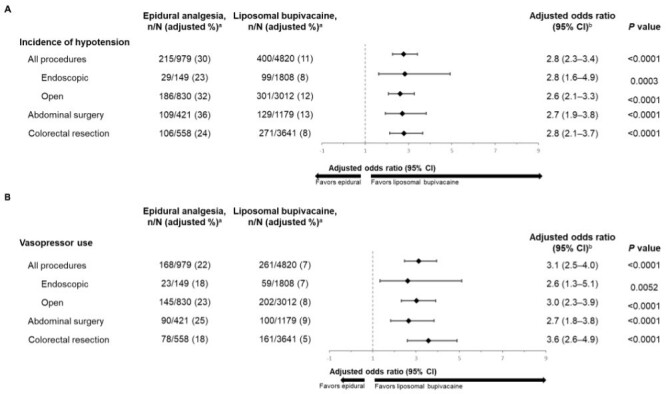
Clinical Outcomes: Incidence of Hypotension (**A**) and Vasopressor Use (**B**) by Procedure Type and Surgery in Patients Given Epidural Analgesia or LB Model adjusted for age, sex, race, Quan-Charlson Comorbidity Index score, insurance type, hospital type, provider region, location, surgical year, bed size, and surgical procedure (all procedures analysis only). Liposomal bupivacaine was used as the reference in odds ratio calculations. Abbreviations: CI, confidence interval; LB, liposomal bupivacaine. ^a^Percentages were rounded to the nearest whole number. ^b^Values were rounded to 1 decimal place.

Across both surgical procedures, patients given epidural analgesia were significantly more likely to experience postoperative hypotension (adjusted rate: 30%) than patients given LB (adjusted rate: 11%; Figure 4A). The significant increase in incidence of postoperative hypotension was also evident when analyzed by approach (endoscopic and open, *P* = .0003 and *P* < .0001, respectively). Epidural analgesia was also significantly associated with higher incidence of hypotension for each surgical procedure (adjusted odds ratios: 2.7 for abdominal surgery and 2.8 for colorectal resection; *P* < .0001 for both).

Patients given epidural analgesia were also more likely to have received postsurgical vasopressors than those given LB across both surgical procedures analyzed (adjusted rate: 22% vs 7%) and for each approach (endoscopic or open; Figure 4B). Epidural analgesia was also significantly associated with postsurgical vasopressor use in each individual surgical procedure (adjusted odds ratios: 2.7 for abdominal surgery and 3.6 for colorectal resection surgery; *P* < .0001 for both).

Given the potential impact of vasopressor use on pharmacy costs, we compared pharmacy costs by vasopressor use (yes or no) in a separate subgroup analysis. Among patients with no vasopressor use (ie, free of incidental hypotension), pharmacy costs associated with epidural analgesia were significantly lower than LB (adjusted costs, $2404 and $2742, respectively; adjusted rate ratio [95% CI], 0.88 [0.82-0.94]; *P* < .0001). However, in patients where vasopressors were used, there was no significant difference in adjusted costs between epidural analgesia and LB (adjusted costs, $6011 and $6247, respectively; adjusted rate ratio [95% CI], 0.96 [0.78-1.19]; *P* = .7239).

Because the sample size between epidural and LB groups was imbalanced, a sensitivity analysis was conducted using the propensity score matching method, with the greedy search algorithm and a ratio of 1 patient who received an epidural matched to 1 patient who received LB. Among matched patients (n = 941 each for the epidural and LB analgesia groups), the sensitivity analysis revealed similar outcomes to the original analysis. Specifically, the propensity score–matched ratio for LOS rate, total hospital cost, discharge to home odds, 30-day readmission odds, hypotension odds, and vasopressor odds were 1.25 (95% CI, 1.16-1.35), 1.25 (95% CI, 1.15-1.36), 0.66 (95% CI, 0.52-0.82), 1.42 (95% CI, 1.06-1.92), 2.48 (95% CI, 1.91-3.23), and 2.81 (95% CI, 2.07-3.81), respectively.

## DISCUSSION

In the current analysis, compared with epidural analgesia, LB was associated with lower LOS and hospital costs and increased rates of discharge to home for both abdominal and colorectal resection surgeries, regardless of surgical approach (open or endoscopic). Additionally, epidural analgesia was associated with higher rates of 30-day hospital readmissions in the overall cohort, which appeared to be driven by the abdominal surgery and endoscopic surgery subgroups. Increased rates of postsurgical hypotension and vasopressor use were associated with epidural analgesia regardless of surgery site or surgical approach. These data suggest LB is associated with reduced healthcare resource utilization and costs in addition to reduced postsurgical hypotension compared with epidural analgesia for both abdominal surgery and colorectal resection. Our finding that patients who received epidural analgesia were hospitalized 1.6 days longer than those who received LB is consistent with another retrospective analysis of approximately 12 000 patients recovering from abdominal surgery, which reported that hospital LOS was approximately 2 days shorter in patients who had TAP blocks than in those given continuous epidural analgesia.^14^ Additionally, the increases in total and itemized costs associated with epidural analgesia compared with LB in the current analysis are in agreement with a retrospective cohort study of 190 patients who had video-assisted thoracoscopic (VATS)–pulmonary resections, which concluded that patients given epidural analgesia had higher total and direct costs than those given LB.^25^ Consistent with LOS trends in the current study, cost savings with LB were largely attributable to the reduced hospital room and board costs, although additional savings in anesthesia and professional fees were also observed. In the current analysis, medical cost savings were observed in not only room and board, anesthesia, and professional fee costs, but also blood bank and respiratory support. Ultimately, higher hospital costs with epidural analgesia may be in part due to the high failure rate of this method, which reportedly ranges from 10% to 30%^26–28^ and is largely attributed to incorrect catheter placement, catheter dislodgement, and challenges due to patient anatomy.^28,29^ Epidural analgesia is also associated with severe complications including but not limited to epidural hematomas, respiratory depression, and postdural puncture headache,^29,30^ although these events are generally rare and therefore likely play a minimal role in overall economic burden.^31,32^

In a secondary analysis, we found that postoperative hypotension incidence and vasopressor use were approximately 2 to 4 times more common with epidural analgesia than with peripheral nerve blocks with LB across both surgical procedures and when assessed individually. These results are generally consistent with a recent multicenter randomized trial (EXPLANE) in which 498 patients recovering from abdominal surgery were randomized to epidural analgesia or TAP block with LB.^33^ In EXPLANE, postoperative hypotension (defined in the trial as any mean arterial pressure <65 mm Hg) was observed in 48% of participants randomized to the epidural group and in 31% of participants randomized to TAP block with LB, with an estimated relative risk of 0.64 (*P* = .006). Another study of patients treated for traumatic rib fractures similarly found that complications (including hypotension and catheter dislodgement, breakage, and nonfunction) were reported in 15 of 58 patients (26%) given epidural analgesia, but no complications were reported in 230 patients given intercostal nerve blocks.^34^ Three recent meta-analyses of clinical trials also reported elevated risk for hypotension resulting from use of epidural analgesia in patients recovering from abdominal and cardiac procedures.^1,11,35^ Increased hypotension risk may also contribute to the increased cost of epidural relative to alternatives because of the need for continued monitoring and care. Of note, the subgroup analysis of patients with and without vasopressor use in the current study suggested that the pharmacy savings associated with the use of epidural analgesia compared with LB were offset by other, much higher medical expenditures.

The benefits of fascial plane blocks should ultimately be weighed against all available options. For example, IV PCA is more cost-effective than either epidural analgesia or TAP blocks with LB after accounting for LOS and opioid-related adverse events,^36^ although the technique may be prone to user errors.^37^ Trials specifically assessing economic outcomes with epidural analgesia, peripheral nerve blocks, and IV PCA would help identify causes of economic differences and allow direct comparisons to efficacy, which are necessary for clinical decision-making. The role of these analgesics in postsurgical multimodal pain management, to optimize pain management while considering economic and clinical outcomes, should be further examined in future studies. Additionally, because this analysis included data only from abdominal surgeries and colorectal resections, additional studies may be warranted to confirm these findings in other surgical populations receiving LB vs epidural analgesia.

### Limitations

The limitations of our analysis are primarily related to the data available in the Premier Healthcare Database. First, medical cost savings associated with the use of LB were largely attributed to the costs of room and board in this analysis. The use of LB was also associated with decreased LOS compared with epidural analgesia. While it is reasonable to conclude that a longer hospital stay would incur more costs, information related to the location of care (eg, floor vs unit) that may contribute to cost differences in room and board was not available. Second, both epidural analgesia and fascial plane blocks with LB may be underreported because bundled payments provide little incentive for hospitals to identify treatment for which they will not be separately reimbursed. Third, codes available in the Premier Healthcare Database cannot confirm whether LB was administered via surgical infiltration or fascial plane blocks. It is also of note that analgesic efficacy was not analyzed, which would be important to weigh against the risk of hypotension and hospital costs. For example, the recent EXPLANE trial including patients undergoing major abdominal surgery showed that pain scores at rest during the initial 3 days after surgery were noninferior with TAP block with LB compared with epidural analgesia; patients who received TAP block with LB consumed an average of 7 mg/day more opioids, although the authors concluded this difference was not clinically meaningful.^33^ Other retrospective analyses report comparable or improved analgesia in patients given LB or epidural analgesia after lower abdominal surgery and VATS.^14,25^ Nevertheless, it remains unclear whether the higher cost of care and hypotension incidence associated with epidural analgesia would justify potential improvements in pain control compared with LB. In the present analysis, we controlled for numerous covariates showing differences between the 2 analgesia groups in the statistical models (Table 1). Subgroup analysis according to covariates also suggested little impact on the results in the multivariable models. For instance, total hospital cost outcomes remain the same in the strata of location and region and in most categories of bed size (*P* ≤ .02). We acknowledge that other variables not available in the data might have confounded the results (ie, residual confounding). For instance, prior pain management might have affected outcomes of hospital LOS and, thus, total hospital costs. Another limitation is the potential bias in the source of cost data from the cost-to-charge ratio conversion. According to the Premier Healthcare Database White Paper, cost data are derived either from the cost-to-charge ratio conversion or from hospital cost accounting systems.^16^ As such, we performed an additional sensitivity analysis by including only patients with cost data directly from the hospital (n = 4409) and observed very similar results, with the new total hospital cost of $31 542 and $24 998 in patients receiving epidural and LB, respectively. Our data are also supported by another study with actual cost data in patients undergoing VATS resection, where there was a mean difference in total hospital cost of $2906 between patients receiving epidural and LB, which was similar to what we observed in our study (ie, a mean difference of $2776 in the endoscopic approach).^25^ Future studies are warranted to confirm our findings.

An additional potential study limitation is the possibility of type I error due to the number of comparisons in the analysis (ie, 30 comparisons for the 6 primary and secondary outcomes). When a more stringent α level based on Bonferroni correction (α = .0017) was applied, most primary outcomes remained statistically significant except for 30-day readmission, which was only marginally significant (*P* = .007). Most results for the subgroup analysis according to surgery procedure and approach also remained significant, with the exception of a few outcomes in the abdominal surgery and endoscopic approaches, where fewer patients were available. Because the conclusion for most primary outcomes still held after controlling for family-wise type I error, we believe that type I error did not substantially impact the conclusions from the current analysis.

Finally, the Premier Healthcare Database does not include blood pressure measures. We therefore report hypotension as an incidence based on clinician diagnosis codes rather than by severity, which would be more clinically meaningful. We similarly report vasopressor use rather than quantities and duration of vasopressor medication.

## CONCLUSIONS

In summary, the costs of epidural analgesia relative to alternative postsurgical analgesia approaches remain poorly appreciated. Our analysis found that epidural analgesia is associated with prolonged hospitalization and increased hospital costs relative to LB use in surgical infiltration or fascial plane blocks in both endoscopic and open abdominal surgeries, including colorectal resection surgeries. Additionally, epidural analgesia was associated with increased risk of hypotension and vasopressor use compared with LB analgesia. The findings support the use of LB as an alternative regional analgesia method to epidural in patients undergoing abdominal surgeries.

### Author Contributions

M.H. and C.V.A. contributed to the study design and analysis and interpretation of data, critically revised the manuscript for intellectual content, and provided final approval of the version to be published, and agreed to be accountable for all aspects of the work. N.L. and J.H.L. contributed to the study design and analysis and interpretation of data, critically revised the manuscript for intellectual content, provided final approval of the version to be published, and agreed to be accountable for all aspects of the work.

### Disclosures

M.H. has received consulting fees and honoraria from Flexion Therapeutics, Pacira Biosciences Inc, and Pajunk Medical Systems. N.L. is a former consultant for Pacira Biosciences Inc. J.H.L. is an employee of Pacira Biosciences, Inc, and may own stock or stock options in the company. C.V.A. has nothing to disclose.

### Data Sharing

Data for this study were available to the authors via third-party license from the Premier Healthcare Database. Pacira Biosciences Inc had the license while performing the analysis of the deidentified data. Researchers may access the data by purchase through the Premier Healthcare Database and apply the study cohort ascertainment criteria.

## Supplementary Material

Online Supplementary Material
