# Supplementary material for: Economic Outcomes and Incidence of Postsurgical Hypotension With Liposomal Bupivacaine vs Epidural Analgesia in Abdominal Surgeries
Source: J Health Econ Outcomes Res. 2022 Sep 14;9(2):86–94. doi: 10.36469/001c.37739 (PMC9473799; doi:10.36469/001c.37739)
Supplement: Online Supplementary Material [file jheor_2022_9_2_37739_99891.pdf]

Journal of Health Economics and Outcomes Research

Online Supplementary Material

Economic Outcomes and Incidence of Postsurgical Hypotension With Liposomal Bupivacaine vs Epidural Analgesia in Abdominal Surgeries. *JHEOR*. 2022;9(2):86-94. doi:10.36469/jheor.2022.37739

Table S1: Standard Charge Codes for Abdominal and Colorectal Resection Surgeries

Table S2: Standard Charge Codes for General Anesthesia

Table S3: Standard Charge Codes for Epidural Analgesia

Table S4: Standard Charge Codes for Liposomal Bupivacaine

Table S5: Complete Regression Tables for Economic and Clinical Outcomes

This supplementary material has been provided by the authors to give readers additional information about their work.

This is an open-access article distributed under the terms of the Creative Commons Attribution 4.0 International License (CCBY-4.0). View this license's legal deed at http://creativecommons.org/licenses/by/4.0 and legal code at http://creativecommons.org/licenses/by/4.0/legalcode for more information.

Table S1. Standard Charge Codes for Abdominal and Colorectal Resection Surgeries

Standard Charge Code	Standard Charge Description
Abdominal surgery codes	
0DB80ZZ	EXCISION OF SMALL INTESTINE, OPEN APPROACH
0DN80ZZ	RELEASE SMALL INTESTINE, OPEN APPROACH
0FBG0ZZ	EXCISION OF PANCREAS, OPEN APPROACH
07TP0ZZ	RESECTION OF SPLEEN, OPEN APPROACH
0DU907Z	SUPPLEMENT DUODENUM WITH AUTOLOGOUS TISSUE SUBSTITUTE, OPEN APPROACH
0DNW0ZZ	RELEASE PERITONEUM, OPEN APPROACH
0DT80ZZ	RESECTION OF SMALL INTESTINE, OPEN APPROACH
0DBA0ZZ	EXCISION OF JEJUNUM, OPEN APPROACH
0DNW4ZZ	RELEASE PERITONEUM, PERCUTANEOUS ENDOSCOPIC APPROACH
0WJP0ZZ	INSPECTION OF GASTROINTESTINAL TRACT, OPEN APPROACH
0DN84ZZ	RELEASE SMALL INTESTINE, PERCUTANEOUS ENDOSCOPIC APPROACH
0DQ60ZZ	REPAIR STOMACH, OPEN APPROACH
0DQ90ZZ	REPAIR DUODENUM, OPEN APPROACH
0WBF0ZZ	EXCISION OF ABDOMINAL WALL, OPEN APPROACH
0DQ80ZZ	REPAIR SMALL INTESTINE, OPEN APPROACH
0FBG4ZZ	EXCISION OF PANCREAS, PERCUTANEOUS ENDOSCOPIC APPROACH
0WJP4ZZ	INSPECTION OF GASTROINTESTINAL TRACT, PERCUTANEOUS ENDOSCOPIC APPROACH
0DB98ZX	EXCISION OF DUODENUM, VIA NATURAL OR ARTIFICIAL OPENING ENDOSCOPIC, DIAGNOSTIC
0DB84ZZ	EXCISION OF SMALL INTESTINE, PERCUTANEOUS ENDOSCOPIC APPROACH
0DB90ZZ	EXCISION OF DUODENUM, OPEN APPROACH
0W3P8ZZ	CONTROL BLEEDING IN GASTROINTESTINAL TRACT, VIA NATURAL OR ARTIFICIAL OPENING ENDOSCOPIC
0DQ70ZZ	REPAIR STOMACH, PYLORUS, OPEN APPROACH
0DBW0ZZ	EXCISION OF PERITONEUM, OPEN APPROACH
07TP4ZZ	RESECTION OF SPLEEN, PERCUTANEOUS ENDOSCOPIC APPROACH
0DT60ZZ	RESECTION OF STOMACH, OPEN APPROACH
0FTG0ZZ	RESECTION OF PANCREAS, OPEN APPROACH
0WBH0ZZ	EXCISION OF RETROPERITONEUM, OPEN APPROACH
0FBG0ZX	EXCISION OF PANCREAS, OPEN APPROACH, DIAGNOSTIC
0DTA0ZZ	RESECTION OF JEJUNUM, OPEN APPROACH
0DNS0ZZ	RELEASE GREATER OMENTUM, OPEN APPROACH
0DNA0ZZ	RELEASE JEJUNUM, OPEN APPROACH
0W0F0ZZ	ALTERATION OF ABDOMINAL WALL, OPEN APPROACH
0DQV4ZZ	REPAIR MESENTERY, PERCUTANEOUS ENDOSCOPIC APPROACH
0DNS4ZZ	RELEASE GREATER OMENTUM, PERCUTANEOUS ENDOSCOPIC APPROACH
0W9F0ZZ	DRAINAGE OF ABDOMINAL WALL, OPEN APPROACH
0GT20ZZ	RESECTION OF LEFT ADRENAL GLAND, OPEN APPROACH
0DQV0ZZ	REPAIR MESENTERY, OPEN APPROACH
0DC80ZZ	EXTIRPATION OF MATTER FROM SMALL INTESTINE, OPEN APPROACH
0GT34ZZ	RESECTION OF RIGHT ADRENAL GLAND, PERCUTANEOUS ENDOSCOPIC APPROACH
0GT30ZZ	RESECTION OF RIGHT ADRENAL GLAND, OPEN APPROACH
0DC60ZZ	EXTIRPATION OF MATTER FROM STOMACH, OPEN APPROACH
0DQA0ZZ	REPAIR JEJUNUM, OPEN APPROACH
0DN64ZZ	RELEASE STOMACH, PERCUTANEOUS ENDOSCOPIC APPROACH

Table S1. Standard Charge Codes for Abdominal and Colorectal Resection Surgeries, *cont'd*

Standard Charge Code	Standard Charge Description
0DB78ZX	EXCISION OF STOMACH, PYLORUS, VIA NATURAL OR ARTIFICIAL OPENING ENDOSCOPIC, DIAGNOSTIC
0DT84ZZ	RESECTION OF SMALL INTESTINE, PERCUTANEOUS ENDOSCOPIC APPROACH
0DBS0ZX	EXCISION OF GREATER OMENTUM, OPEN APPROACH, DIAGNOSTIC
0D1A0ZA	BYPASS JEJUNUM TO JEJUNUM, OPEN APPROACH
0D1A0Z4	BYPASS JEJUNUM TO CUTANEOUS, OPEN APPROACH
0WBH0ZX	EXCISION OF RETROPERITONEUM, OPEN APPROACH, DIAGNOSTIC
0WBF0ZX	EXCISION OF ABDOMINAL WALL, OPEN APPROACH, DIAGNOSTIC
0DB80ZX	EXCISION OF SMALL INTESTINE, OPEN APPROACH, DIAGNOSTIC
0DCA0ZZ	EXTIRPATION OF MATTER FROM JEJUNUM, OPEN APPROACH
0DU947Z	SUPPLEMENT DUODENUM WITH AUTOLOGOUS TISSUE SUBSTITUTE, PERCUTANEOUS ENDOSCOPIC APPROACH
0DSA4ZZ	REPOSITION JEJUNUM, PERCUTANEOUS ENDOSCOPIC APPROACH
0DBW0ZX	EXCISION OF PERITONEUM, OPEN APPROACH, DIAGNOSTIC
0DQ98ZZ	REPAIR DUODENUM, VIA NATURAL OR ARTIFICIAL OPENING ENDOSCOPIC
0DQ74ZZ	REPAIR STOMACH, PYLORUS, PERCUTANEOUS ENDOSCOPIC APPROACH
0DQ64ZZ	REPAIR STOMACH, PERCUTANEOUS ENDOSCOPIC APPROACH
0WJP8ZZ	INSPECTION OF GASTROINTESTINAL TRACT, VIA NATURAL OR ARTIFICIAL OPENING ENDOSCOPIC APPROACH
0D20XUZ	CHANGE FEEDING DEVICE IN UPPER INTESTINAL TRACT, EXTERNAL APPROACH
0DB70ZZ	EXCISION OF STOMACH, PYLORUS, OPEN APPROACH
0DHA3UZ	INSERTION OF FEEDING DEVICE INTO JEJUNUM, PERCUTANEOUS APPROACH
0F1G0Z3	BYPASS PANCREAS TO DUODENUM, OPEN APPROACH
0DHA0UZ	INSERTION OF FEEDING DEVICE INTO JEJUNUM, OPEN APPROACH
0DT90ZZ	RESECTION OF DUODENUM, OPEN APPROACH
0F9G0ZZ	DRAINAGE OF PANCREAS, OPEN APPROACH
0DT70ZZ	RESECTION OF STOMACH, PYLORUS, OPEN APPROACH
0D874ZZ	DIVISION OF STOMACH, PYLORUS, PERCUTANEOUS ENDOSCOPIC APPROACH
0DQ84ZZ	REPAIR SMALL INTESTINE, PERCUTANEOUS ENDOSCOPIC APPROACH
07BP0ZZ	EXCISION OF SPLEEN, OPEN APPROACH
0DQ68ZZ	REPAIR STOMACH, VIA NATURAL OR ARTIFICIAL OPENING ENDOSCOPIC
0DBA0ZX	EXCISION OF JEJUNUM, OPEN APPROACH, DIAGNOSTIC
0WJF0ZZ	INSPECTION OF ABDOMINAL WALL, OPEN APPROACH
0DNA4ZZ	RELEASE JEJUNUM, PERCUTANEOUS ENDOSCOPIC APPROACH
0DN90ZZ	RELEASE DUODENUM, OPEN APPROACH
0W9H0ZZ	DRAINAGE OF RETROPERITONEUM, OPEN APPROACH
0W3P0ZZ	CONTROL BLEEDING IN GASTROINTESTINAL TRACT, OPEN APPROACH
07QP0ZZ	REPAIR SPLEEN, OPEN APPROACH
0DNV0ZZ	RELEASE MESENTERY, OPEN APPROACH
0GT24ZZ	RESECTION OF LEFT ADRENAL GLAND, PERCUTANEOUS ENDOSCOPIC APPROACH
0FTG4ZZ	RESECTION OF PANCREAS, PERCUTANEOUS ENDOSCOPIC APPROACH
0DTS0ZZ	RESECTION OF GREATER OMENTUM, OPEN APPROACH
0DHA4UZ	INSERTION OF FEEDING DEVICE INTO JEJUNUM, PERCUTANEOUS ENDOSCOPIC APPROACH
0DU90JZ	SUPPLEMENT DUODENUM WITH SYNTHETIC SUBSTITUTE, OPEN APPROACH
0DJ64ZZ	INSPECTION OF STOMACH, PERCUTANEOUS ENDOSCOPIC APPROACH

Table S1. Standard Charge Codes for Abdominal and Colorectal Resection Surgeries, *cont'd*

Standard Charge Code	Standard Charge Description
0DJW0ZZ	INSPECTION OF PERITONEUM, OPEN APPROACH
07BP4ZZ	EXCISION OF SPLEEN, PERCUTANEOUS ENDOSCOPIC APPROACH
0DQA4ZZ	REPAIR JEJUNUM, PERCUTANEOUS ENDOSCOPIC APPROACH
0DBS4ZZ	EXCISION OF GREATER OMENTUM, PERCUTANEOUS ENDOSCOPIC APPROACH
0DQ94ZZ	REPAIR DUODENUM, PERCUTANEOUS ENDOSCOPIC APPROACH
0DBS0ZZ	EXCISION OF GREATER OMENTUM, OPEN APPROACH
0DBA4ZZ	EXCISION OF JEJUNUM, PERCUTANEOUS ENDOSCOPIC APPROACH
0WJH0ZZ	INSPECTION OF RETROPERITONEUM, OPEN APPROACH
0D568ZZ	DESTRUCTION OF STOMACH, VIA NATURAL OR ARTIFICIAL OPENING ENDOSCOPIC
0DN83ZZ	RELEASE SMALL INTESTINE, PERCUTANEOUS APPROACH
0KBK0ZX	EXCISION OF RIGHT ABDOMEN MUSCLE, OPEN APPROACH, DIAGNOSTIC
0GB24ZZ	EXCISION OF LEFT ADRENAL GLAND, PERCUTANEOUS ENDOSCOPIC APPROACH
0DL90ZZ	OCCLUSION OF DUODENUM, OPEN APPROACH
0GB20ZZ	EXCISION OF LEFT ADRENAL GLAND, OPEN APPROACH
0D1A4ZA	BYPASS JEJUNUM TO JEJUNUM, PERCUTANEOUS ENDOSCOPIC APPROACH
0FJG0ZZ	INSPECTION OF PANCREAS, OPEN APPROACH
0DJ04ZZ	INSPECTION OF UPPER INTESTINAL TRACT, PERCUTANEOUS ENDOSCOPIC APPROACH
0F9G4ZZ	DRAINAGE OF PANCREAS, PERCUTANEOUS ENDOSCOPIC APPROACH
0F9G3ZZ	DRAINAGE OF PANCREAS, PERCUTANEOUS APPROACH
0DVA4ZZ	RESTRICTION OF JEJUNUM, PERCUTANEOUS ENDOSCOPIC APPROACH
0D160Z4	BYPASS STOMACH TO CUTANEOUS, OPEN APPROACH
0D1A4Z4	BYPASS JEJUNUM TO CUTANEOUS, PERCUTANEOUS ENDOSCOPIC APPROACH
0D9W0ZX	DRAINAGE OF PERITONEUM, OPEN APPROACH, DIAGNOSTIC
0DCW0ZZ	EXTIRPATION OF MATTER FROM PERITONEUM, OPEN APPROACH
0DW60MZ	REVISION OF STIMULATOR LEAD IN STOMACH, OPEN APPROACH
0D9A40Z	DRAINAGE OF JEJUNUM WITH DRAINAGE DEVICE, PERCUTANEOUS ENDOSCOPIC APPROACH
0DC90ZZ	EXTIRPATION OF MATTER FROM DUODENUM, OPEN APPROACH
0D9W4ZZ	DRAINAGE OF PERITONEUM, PERCUTANEOUS ENDOSCOPIC APPROACH
0DC84ZZ	EXTIRPATION OF MATTER FROM SMALL INTESTINE, PERCUTANEOUS ENDOSCOPIC APPROACH
0D1A0ZB	BYPASS JEJUNUM TO ILEUM, OPEN APPROACH
0D9A30Z	DRAINAGE OF JEJUNUM WITH DRAINAGE DEVICE, PERCUTANEOUS APPROACH
0DT64ZZ	RESECTION OF STOMACH, PERCUTANEOUS ENDOSCOPIC APPROACH
0DC68ZZ	EXTIRPATION OF MATTER FROM STOMACH, VIA NATURAL OR ARTIFICIAL OPENING ENDOSCOPIC
0DSA8ZZ	REPOSITION JEJUNUM, VIA NATURAL OR ARTIFICIAL OPENING ENDOSCOPIC
0DB74ZZ	EXCISION OF STOMACH, PYLORUS, PERCUTANEOUS ENDOSCOPIC APPROACH
0GB23ZZ	EXCISION OF LEFT ADRENAL GLAND, PERCUTANEOUS APPROACH
0D9880Z	DRAINAGE OF SMALL INTESTINE WITH DRAINAGE DEVICE, VIA NATURAL OR ARTIFICIAL OPENING ENDOSCOPIC
0DC64ZZ	EXTIRPATION OF MATTER FROM STOMACH, PERCUTANEOUS ENDOSCOPIC APPROACH
0DSA0ZZ	REPOSITION JEJUNUM, OPEN APPROACH
0DS64ZZ	REPOSITION STOMACH, PERCUTANEOUS ENDOSCOPIC APPROACH
0DS60ZZ	REPOSITION STOMACH, OPEN APPROACH
0D964ZZ	DRAINAGE OF STOMACH, PERCUTANEOUS ENDOSCOPIC APPROACH
0DQW0ZZ	REPAIR PERITONEUM, OPEN APPROACH

Table S1. Standard Charge Codes for Abdominal and Colorectal Resection Surgeries, *cont'd*

Standard Charge Code	Standard Charge Description
0D960ZX	DRAINAGE OF STOMACH, OPEN APPROACH, DIAGNOSTIC
0DBT0ZZ	EXCISION OF LESSER OMENTUM, OPEN APPROACH
0D870ZZ	DIVISION OF STOMACH, PYLORUS, OPEN APPROACH
0DP04CZ	REMOVAL OF EXTRALUMINAL DEVICE FROM UPPER INTESTINAL TRACT, PERCUTANEOUS ENDOSCOPIC APPROACH
0D190ZA	BYPASS DUODENUM TO JEJUNUM, OPEN APPROACH
0D798ZZ	DILATION OF DUODENUM, VIA NATURAL OR ARTIFICIAL OPENING ENDOSCOPIC
0DNW3ZZ	RELEASE PERITONEUM, PERCUTANEOUS APPROACH
0DNV4ZZ	RELEASE MESENTERY, PERCUTANEOUS ENDOSCOPIC APPROACH
0D778ZZ	DILATION OF STOMACH, PYLORUS, VIA NATURAL OR ARTIFICIAL OPENING ENDOSCOPIC
0WWP0YZ	REVISION OF OTHER DEVICE IN GASTROINTESTINAL TRACT, OPEN APPROACH
0DNT0ZZ	RELEASE LESSER OMENTUM, OPEN APPROACH
0DB90ZX	EXCISION OF DUODENUM, OPEN APPROACH, DIAGNOSTIC
0WBH4ZZ	EXCISION OF RETROPERITONEUM, PERCUTANEOUS ENDOSCOPIC APPROACH
0D190Z4	BYPASS DUODENUM TO CUTANEOUS, OPEN APPROACH
0D760ZZ	DILATION OF STOMACH, OPEN APPROACH
0DB88ZX	EXCISION OF SMALL INTESTINE, VIA NATURAL OR ARTIFICIAL OPENING ENDOSCOPIC, DIAGNOSTIC
0DN87ZZ	RELEASE SMALL INTESTINE, VIA NATURAL OR ARTIFICIAL OPENING
0W3P4ZZ	CONTROL BLEEDING IN GASTROINTESTINAL TRACT, PERCUTANEOUS ENDOSCOPIC APPROACH
0W3H0ZZ	CONTROL BLEEDING IN RETROPERITONEUM, OPEN APPROACH
0KQK0ZZ	REPAIR RIGHT ABDOMEN MUSCLE, OPEN APPROACH
047H3EZ	DILATION OF RIGHT EXTERNAL ILIAC ARTERY WITH TWO INTRALUMINAL DEVICES, PERCUTANEOUS APPROACH
0D160ZL	BYPASS STOMACH TO TRANSVERSE COLON, OPEN APPROACH
0KBL0ZZ	EXCISION OF LEFT ABDOMEN MUSCLE, OPEN APPROACH
0KBK0ZZ	EXCISION OF RIGHT ABDOMEN MUSCLE, OPEN APPROACH
0DN74ZZ	RELEASE STOMACH, PYLORUS, PERCUTANEOUS ENDOSCOPIC APPROACH
0DN60ZZ	RELEASE STOMACH, OPEN APPROACH
**Colorectal Resection Codes**	
0DTF0ZZ	RESECTION OF RIGHT LARGE INTESTINE, OPEN APPROACH
0DTN0ZZ	RESECTION OF SIGMOID COLON, OPEN APPROACH
0DBN0ZZ	EXCISION OF SIGMOID COLON, OPEN APPROACH
0DTF4ZZ	RESECTION OF RIGHT LARGE INTESTINE, PERCUTANEOUS ENDOSCOPIC APPROACH
0DBB0ZZ	EXCISION OF ILEUM, OPEN APPROACH
0DTN4ZZ	RESECTION OF SIGMOID COLON, PERCUTANEOUS ENDOSCOPIC APPROACH
0DBN4ZZ	EXCISION OF SIGMOID COLON, PERCUTANEOUS ENDOSCOPIC APPROACH
0DBP4ZZ	EXCISION OF RECTUM, PERCUTANEOUS ENDOSCOPIC APPROACH
0DTG0ZZ	RESECTION OF LEFT LARGE INTESTINE, OPEN APPROACH
0DTP4ZZ	RESECTION OF RECTUM, PERCUTANEOUS ENDOSCOPIC APPROACH
0DBP0ZZ	EXCISION OF RECTUM, OPEN APPROACH
0DTP0ZZ	RESECTION OF RECTUM, OPEN APPROACH
0DBL0ZZ	EXCISION OF TRANSVERSE COLON, OPEN APPROACH
0DBM0ZZ	EXCISION OF DESCENDING COLON, OPEN APPROACH
0D1B0Z4	BYPASS ILEUM TO CUTANEOUS, OPEN APPROACH
0D1B4Z4	BYPASS ILEUM TO CUTANEOUS, PERCUTANEOUS ENDOSCOPIC APPROACH

Table S1. Standard Charge Codes for Abdominal and Colorectal Resection Surgeries, *cont'd*

Standard Charge Code	Standard Charge Description
0DTE0ZZ	RESECTION OF LARGE INTESTINE, OPEN APPROACH
0DTG4ZZ	RESECTION OF LEFT LARGE INTESTINE, PERCUTANEOUS ENDOSCOPIC APPROACH
0DBE0ZZ	EXCISION OF LARGE INTESTINE, OPEN APPROACH
0DTH0ZZ	RESECTION OF CECUM, OPEN APPROACH
0DTE4ZZ	RESECTION OF LARGE INTESTINE, PERCUTANEOUS ENDOSCOPIC APPROACH
0D1L0Z4	BYPASS TRANSVERSE COLON TO CUTANEOUS, OPEN APPROACH
0D1N4Z4	BYPASS SIGMOID COLON TO CUTANEOUS, PERCUTANEOUS ENDOSCOPIC APPROACH
0DTK4ZZ	RESECTION OF ASCENDING COLON, PERCUTANEOUS ENDOSCOPIC APPROACH
0D1N0Z4	BYPASS SIGMOID COLON TO CUTANEOUS, OPEN APPROACH
0DBK4ZZ	EXCISION OF ASCENDING COLON, PERCUTANEOUS ENDOSCOPIC APPROACH
0DTK0ZZ	RESECTION OF ASCENDING COLON, OPEN APPROACH
0DBM4ZZ	EXCISION OF DESCENDING COLON, PERCUTANEOUS ENDOSCOPIC APPROACH
0DBH4ZZ	EXCISION OF CECUM, PERCUTANEOUS ENDOSCOPIC APPROACH
0DTL0ZZ	RESECTION OF TRANSVERSE COLON, OPEN APPROACH
0DTH4ZZ	RESECTION OF CECUM, PERCUTANEOUS ENDOSCOPIC APPROACH
0DBF0ZZ	EXCISION OF RIGHT LARGE INTESTINE, OPEN APPROACH
0DBL4ZZ	EXCISION OF TRANSVERSE COLON, PERCUTANEOUS ENDOSCOPIC APPROACH
0DBH0ZZ	EXCISION OF CECUM, OPEN APPROACH
0D1M0Z4	BYPASS DESCENDING COLON TO CUTANEOUS, OPEN APPROACH
0DBB4ZZ	EXCISION OF ILEUM, PERCUTANEOUS ENDOSCOPIC APPROACH
0DBF4ZZ	EXCISION OF RIGHT LARGE INTESTINE, PERCUTANEOUS ENDOSCOPIC APPROACH
0DBK0ZZ	EXCISION OF ASCENDING COLON, OPEN APPROACH
0DTL4ZZ	RESECTION OF TRANSVERSE COLON, PERCUTANEOUS ENDOSCOPIC APPROACH
0DTM0ZZ	RESECTION OF DESCENDING COLON, OPEN APPROACH
0DTB0ZZ	RESECTION OF ILEUM, OPEN APPROACH
0DBP7ZZ	EXCISION OF RECTUM, VIA NATURAL OR ARTIFICIAL OPENING
0D1M4Z4	BYPASS DESCENDING COLON TO CUTANEOUS, PERCUTANEOUS ENDOSCOPIC APPROACH
0DBE4ZZ	EXCISION OF LARGE INTESTINE, PERCUTANEOUS ENDOSCOPIC APPROACH
0DTB4ZZ	RESECTION OF ILEUM, PERCUTANEOUS ENDOSCOPIC APPROACH
0DBG0ZZ	EXCISION OF LEFT LARGE INTESTINE, OPEN APPROACH
0DBN8ZX	EXCISION OF SIGMOID COLON, VIA NATURAL OR ARTIFICIAL OPENING ENDOSCOPIC, DIAGNOSTIC
0D1L4Z4	BYPASS TRANSVERSE COLON TO CUTANEOUS, PERCUTANEOUS ENDOSCOPIC APPROACH
0DBK8ZX	EXCISION OF ASCENDING COLON, VIA NATURAL OR ARTIFICIAL OPENING ENDOSCOPIC, DIAGNOSTIC
0DBP8ZX	EXCISION OF RECTUM, VIA NATURAL OR ARTIFICIAL OPENING ENDOSCOPIC, DIAGNOSTIC
0DBP8ZZ	EXCISION OF RECTUM, VIA NATURAL OR ARTIFICIAL OPENING ENDOSCOPIC
0D1N0ZP	BYPASS SIGMOID COLON TO RECTUM, OPEN APPROACH
0D1B0ZB	BYPASS ILEUM TO ILEUM, OPEN APPROACH
0D1B0ZL	BYPASS ILEUM TO TRANSVERSE COLON, OPEN APPROACH
0D1B0ZQ	BYPASS ILEUM TO ANUS, OPEN APPROACH
0DBG4ZZ	EXCISION OF LEFT LARGE INTESTINE, PERCUTANEOUS ENDOSCOPIC APPROACH
0DTM4ZZ	RESECTION OF DESCENDING COLON, PERCUTANEOUS ENDOSCOPIC APPROACH
0DBN0ZX	EXCISION OF SIGMOID COLON, OPEN APPROACH, DIAGNOSTIC
0D1B4ZQ	BYPASS ILEUM TO ANUS, PERCUTANEOUS ENDOSCOPIC APPROACH

Table S1. Standard Charge Codes for Abdominal and Colorectal Resection Surgeries, *cont'd*

Standard Charge Code	Standard Charge Description
0DBM8ZX	EXCISION OF DESCENDING COLON, VIA NATURAL OR ARTIFICIAL OPENING ENDOSCOPIC, DIAGNOSTIC
0DBP0ZX	EXCISION OF RECTUM, OPEN APPROACH, DIAGNOSTIC
0DBP7ZX	EXCISION OF RECTUM, VIA NATURAL OR ARTIFICIAL OPENING, DIAGNOSTIC
0DTC4ZZ	RESECTION OF ILEOCECAL VALVE, PERCUTANEOUS ENDOSCOPIC APPROACH
0DTP7ZZ	RESECTION OF RECTUM, VIA NATURAL OR ARTIFICIAL OPENING
0D1B0J4	BYPASS ILEUM TO CUTANEOUS WITH SYNTHETIC SUBSTITUTE, OPEN APPROACH
0D1B4ZH	BYPASS ILEUM TO CECUM, PERCUTANEOUS ENDOSCOPIC APPROACH
0D1M0ZP	BYPASS DESCENDING COLON TO RECTUM, OPEN APPROACH
0D1M4ZP	BYPASS DESCENDING COLON TO RECTUM, PERCUTANEOUS ENDOSCOPIC APPROACH
0DBH8ZZ	EXCISION OF CECUM, VIA NATURAL OR ARTIFICIAL OPENING ENDOSCOPIC
0DBL8ZX	EXCISION OF TRANSVERSE COLON, VIA NATURAL OR ARTIFICIAL OPENING ENDOSCOPIC, DIAGNOSTIC
0DBN3ZZ	EXCISION OF SIGMOID COLON, PERCUTANEOUS APPROACH
0DTP8ZZ	RESECTION OF RECTUM, VIA NATURAL OR ARTIFICIAL OPENING ENDOSCOPIC
0D1B074	BYPASS ILEUM TO CUTANEOUS WITH AUTOLOGOUS TISSUE SUBSTITUTE, OPEN APPROACH
0D1B07B	BYPASS ILEUM TO ILEUM WITH AUTOLOGOUS TISSUE SUBSTITUTE, OPEN APPROACH
0D1B07L	BYPASS ILEUM TO TRANSVERSE COLON WITH AUTOLOGOUS TISSUE SUBSTITUTE, OPEN APPROACH
0D1B0KB	BYPASS ILEUM TO ILEUM WITH NONAUTOLOGOUS TISSUE SUBSTITUTE, OPEN APPROACH
0D1B0ZH	BYPASS ILEUM TO CECUM, OPEN APPROACH
0D1B3J4	BYPASS ILEUM TO CUTANEOUS WITH SYNTHETIC SUBSTITUTE, PERCUTANEOUS APPROACH
0D1B4ZL	BYPASS ILEUM TO TRANSVERSE COLON, PERCUTANEOUS ENDOSCOPIC APPROACH
0D1B4ZM	BYPASS ILEUM TO DESCENDING COLON, PERCUTANEOUS ENDOSCOPIC APPROACH
0D1H0Z4	BYPASS CECUM TO CUTANEOUS, OPEN APPROACH
0D1H0ZP	BYPASS CECUM TO RECTUM, OPEN APPROACH
0D1K0Z4	BYPASS ASCENDING COLON TO CUTANEOUS, OPEN APPROACH
0D1K4Z4	BYPASS ASCENDING COLON TO CUTANEOUS, PERCUTANEOUS ENDOSCOPIC APPROACH
0D1K4ZP	BYPASS ASCENDING COLON TO RECTUM, PERCUTANEOUS ENDOSCOPIC APPROACH
0D1L0ZP	BYPASS TRANSVERSE COLON TO RECTUM, OPEN APPROACH
0D1M07P	BYPASS DESCENDING COLON TO RECTUM WITH AUTOLOGOUS TISSUE SUBSTITUTE, OPEN APPROACH
0D1M0J4	BYPASS DESCENDING COLON TO CUTANEOUS WITH SYNTHETIC SUBSTITUTE, OPEN APPROACH
0D1M4J4	BYPASS DESCENDING COLON TO CUTANEOUS WITH SYNTHETIC SUBSTITUTE, PERCUTANEOUS ENDOSCOPIC APPROACH
0D1N074	BYPASS SIGMOID COLON TO CUTANEOUS WITH AUTOLOGOUS TISSUE SUBSTITUTE, OPEN APPROACH
0D1N0ZN	BYPASS SIGMOID COLON TO SIGMOID COLON, OPEN APPROACH
0D1N474	BYPASS SIGMOID COLON TO CUTANEOUS WITH AUTOLOGOUS TISSUE SUBSTITUTE, PERCUTANEOUS ENDOSCOPIC APPROACH
0D1N4J4	BYPASS SIGMOID COLON TO CUTANEOUS WITH SYNTHETIC SUBSTITUTE, PERCUTANEOUS ENDOSCOPIC APPROACH
0D1N4ZP	BYPASS SIGMOID COLON TO RECTUM, PERCUTANEOUS ENDOSCOPIC APPROACH
0DBB0ZX	EXCISION OF ILEUM, OPEN APPROACH, DIAGNOSTIC
0DBB8ZX	EXCISION OF ILEUM, VIA NATURAL OR ARTIFICIAL OPENING ENDOSCOPIC, DIAGNOSTIC
0DBH0ZX	EXCISION OF CECUM, OPEN APPROACH, DIAGNOSTIC
0DBK0ZX	EXCISION OF ASCENDING COLON, OPEN APPROACH, DIAGNOSTIC

Table S1. Standard Charge Codes for Abdominal and Colorectal Resection Surgeries, *cont'd*

Standard Charge Code	Standard Charge Description
0DBN8ZZ	EXCISION OF SIGMOID COLON, VIA NATURAL OR ARTIFICIAL OPENING ENDOSCOPIC
0DBP3ZZ	EXCISION OF RECTUM, PERCUTANEOUS APPROACH
0DTN8ZZ	RESECTION OF SIGMOID COLON, VIA NATURAL OR ARTIFICIAL OPENING ENDOSCOPIC
0DXE0Z5	TRANSFER LARGE INTESTINE TO ESOPHAGUS, OPEN APPROACH

Table S2. Standard Charge Codes for General Anesthesia

Standard Charge Code	Standard Charge Description
250250001490000	ALFENTANIL, ALFENTA AMP 500MCG/ML 10ML
250250001500000	ALFENTANIL, ALFENTA AMP 500MCG/ML 1ML
250250001510000	ALFENTANIL, ALFENTA AMP 500MCG/ML 20ML
250250001520000	ALFENTANIL, ALFENTA AMP 500MCG/ML 2ML
250250001530000	ALFENTANIL, ALFENTA AMP 500MCG/ML 5ML
250250020960000	DROPERIDOL, INAPSINE AMP 2.5MG/ML 1ML
250250020970000	DROPERIDOL, INAPSINE VL 2.5MG/ML 10ML
250250020980000	DROPERIDOL, INAPSINE VL 2.5MG/ML 2ML
250250020990000	DROPERIDOL, INAPSINE VL 2.5MG/ML 5ML
250250025030000	ETOMIDATE, AMIDATE VL 2MG/ML 10ML
250250025040000	ETOMIDATE, AMIDATE VL 2MG/ML 1ML
250250025050000	ETOMIDATE, AMIDATE VL 2MG/ML 20ML
250250035920000	KETAMINE, KETALAR VL 100MG/ML 1ML
250250035930000	KETAMINE, KETALAR VL 100MG/ML 5ML
250250035940000	KETAMINE, KETALAR VL 10MG/ML 1ML
250250035950000	KETAMINE, KETALAR VL 10MG/ML 20ML
250250035960000	KETAMINE, KETALAR VL 10MG/ML 25ML
250250035970000	KETAMINE, KETALAR VL 10MG/ML 50ML
250250035980000	KETAMINE, KETALAR VL 50MG/ML 10ML
250250035990000	KETAMINE, KETALAR VL 50MG/ML 1ML
250250041590000	METHOHEXITAL, BREVITAL VL 100MG 10ML
250250041600000	METHOHEXITAL, BREVITAL VL 10MG 1ML
250250041610000	METHOHEXITAL, BREVITAL VL 2.5GM
250250041620000	METHOHEXITAL, BREVITAL VL 500MG
250250041630000	METHOHEXITAL, BREVITAL VL 5GM
250250055710000	PROPOFOL, DIPRIVAN AMP 10MG/ML 10ML
250250055720000	PROPOFOL, DIPRIVAN AMP 10MG/ML 20ML
250250055730000	PROPOFOL, DIPRIVAN VL 10MG/ML 100ML
250250055740000	PROPOFOL, DIPRIVAN VL 10MG/ML 1ML
250250055750000	PROPOFOL, DIPRIVAN VL 10MG/ML 50ML
250250057200000	REMIFENTANIL, ULTIVA VL 1MG 3ML
250250057210000	REMIFENTANIL, ULTIVA VL 2MG 5ML
250250057220000	REMIFENTANIL, ULTIVA VL 5MG 10ML
250250060010000	SUFENTANIL, SUFENTA AMP 50MCG/ML 1ML
250250060020000	SUFENTANIL, SUFENTA AMP 50MCG/ML 2ML
250250060030000	SUFENTANIL, SUFENTA AMP 50MCG/ML 5ML
250250062040000	THIAMYLAL, SURITAL VL 1GM
250250062050000	THIAMYLAL, SURITAL VL 5GM

Table S2. Standard Charge Codes for General Anesthesia, *cont'd*

Standard Charge Code	Standard Charge Description
250250062100000	THIOPENTAL, PENTOTHAL VL 250MG
250250062110000	THIOPENTAL, PENTOTHAL VL 25MG
250250062120000	THIOPENTAL, PENTOTHAL VL 400MG
250250062140000	THIOPENTAL, PENTOTHAL VL 50MG
250250062150000	THIOPENTAL, PENTOTHAL KIT 1GM
250250062160000	THIOPENTAL, PENTOTHAL KIT 2.5GM
250250062170000	THIOPENTAL, PENTOTHAL KIT 5GM
250250062180000	THIOPENTAL, PENTOTHAL REC SUSP 2GM
250250062190000	THIOPENTAL, PENTOTHAL VL 1GM
250250062200000	THIOPENTAL, PENTOTHAL VL 500MG
250250110890000	FOSPROPOFOL, LUSEDRA VL 35MG/ML 30ML
250250122330000	METHOHEXITAL, BREVITAL VL 200MG
250888000550000	ALFENTANIL PARENTERAL MISC
250888007090000	DROPERIDOL PARENTERAL MISC
250888008210000	ETOMIDATE PARENTERAL MISC
250888009110000	FOSPROPOFOL PARENTERAL MISC
250888011530000	KETAMINE PARENTERAL MISC
250888013430000	METHOHEXITAL PARENTERAL MISC
250888017850000	PROPOFOL PARENTERAL MISC
250888018470000	REMIFENTANIL PARENTERAL MISC
250888019880000	SUFENTANIL PARENTERAL MISC
250888020700000	THIAMYLAL PARENTERAL MISC
250888020750000	THIOPENTAL PARENTERAL MISC
250999000420000	ALFENTANIL MISC
250999005770000	DROPERIDOL MISC
250999006570000	ETOMIDATE MISC
250999009730000	KETAMINE MISC
250999011210000	METHOHEXITAL MISC
250999015010000	PROPOFOL MISC
250999015540000	REMIFENTANIL MISC
250999016710000	SUFENTANIL MISC
250999017390000	THIAMYLAL MISC
250999017430000	THIOPENTAL MISC
250999019640000	FENTANYL/BUPIVACAINE MISC
250999027520000	FOSPROPOFOL MISC
250999029430000	FENTANYL/ROPIVACAINE MISC

Table S3. Standard Charge Codes for Epidural Analgesia

Standard Charge Code	Standard Charge Description
250250026120000	FENTANYL, SUBLIMAZE EPIDURAL 250ML
250250032120000	HYDROMORPHONE, DILAUDID EPIDURAL 250ML
250250040350000	MEPERIDINE, DEMEROL EPIDURAL 250ML
270270001630000	BAG EPIDURAL
270270010060000	CATHETER EPIDURAL DUPENS
270270010070000	CATHETER EPIDURAL L/D ANES
270270010080000	CATHETER EPIDURAL SPECIALTY
270270030750000	KIT NEEDLE EPIDURAL MINI
270270033690000	NEEDLE EPIDURAL
270270033700000	NEEDLE EPIDURAL 20GX2.5
270270033710000	NEEDLE EPIDURAL MINI KIT
270270040040000	SET EPIDURAL
270270052610000	TRAY ANES EPIDURAL
270270053640000	TRAY EPIDURAL CONTINUOUS
270270094360000	PUMP EPIDURAL SETUP
270270095190000	CATHETER EPIDURAL
270270103580000	SET EPIDURAL CATH
270270110420000	SET EPIDURAL PUMP
290290002910000	MONITOR PUMP EPIDURAL
360360622750000	INJ ANES EPIDURAL CERVICAL/THORACIC SINGLE
360360622780000	INJ ANES EPIDURAL LUMBAR/CAUDAL SINGLE
360360622790000	INJ ANES EPIDURAL LUMBAR/CAUDAL CONTINUOUS
360360622890000	INJ EPIDURAL LUMBAR/CAUDAL
360360622980000	INJ EPIDURAL CERVICAL/THORACIC
360360623100000	INJ SINGLE EPID/SUBARACH CERV/THOR
360360623110000	INJ SINGLE EPID/SUBARACH LUMB/SACRAL
360360623180000	INJ W CATH PLACEMENT EPID/SUBARACH CERV/THOR
360360623190000	INJ W CATH PLACEMENT EPID/SUBARACH LUMB/SACRAL
360360623550000	REMOVE INTRATHECAL/EPIDURAL CATHETER
360450622750000	ER INJ ANES EPIDURAL CERVICAL/THORACIC SINGLE
360450622780000	ER INJ ANES EPIDURAL LUMBAR/CAUDAL SINGLE
360450622790000	ER INJ ANES EPIDURAL LUMBAR/CAUDAL CONTINUOUS
360450622890000	ER INJ EPIDURAL LUMBAR/CAUDAL
360450622980000	ER INJ EPIDURAL CERVICAL/THORACIC
360450623100000	ER INJ SINGLE EPID/SUBARACH CERV/THOR
360450623110000	ER INJ SINGLE EPID/SUBARACH LUMB/SACRAL
360450623180000	ER INJ W CATH PLACEMENT EPID/SUBARACH CERV/THOR
360450623190000	ER INJ W CATH PLACEMENT EPID/SUBARACH LUMB/SACRAL
360450623550000	ER REMOVE INTRATHECAL/EPIDURAL CATHETER
360490622750000	INJ ANES EPIDURAL CERVICAL/THORACIC SINGLE OP
360490622780000	INJ ANES EPIDURAL LUMBAR/CAUDAL SINGLE OP
360490622790000	INJ ANES EPIDURAL LUMBAR/CAUDAL CONTINUOUS OP
360490622890000	INJ EPIDURAL LUMBAR/CAUDAL OP
360490622980000	INJ EPIDURAL CERVICAL/THORACIC OP
360490623550000	REMOVE INTRATHECAL/EPIDURAL CATHETER OP
360761623100000	TR INJ SINGLE EPID/SUBARACH CERV/THOR

Table S3. Standard Charge Codes for Epidural Analgesia, *cont'd*

Standard Charge Code	Standard Charge Description
360761623110000	TR INJ SINGLE EPID/SUBARACH LUMB/SACRAL
360761623180000	TR INJ W CATH PLACEMENT EPID/SUBARACH CERV/THOR
360761623190000	TR INJ W CATH PLACEMENT EPID/SUBARACH LUMB/SACRAL
360761623550000	TR REMOVE INTRATHECAL/EPIDURAL CATHETER
370370000700000	ANES EPIDURAL ADDL 30 MIN
370370000730000	ANES EPIDURAL FLAT RATE
370370000740000	ANES EPIDURAL 1ST 30 MIN
370370000750000	ANES CRNA EPIDURAL FLAT RATE
370370001440000	ANES EPIDURAL 1ST HR
370370001440001	ANES EPIDURAL 1 HR
370370001450000	ANES EPIDURAL 2 HR
370370001460000	ANES EPIDURAL 2 HR 30 MIN
370370001470000	ANES EPIDURAL 3 HR
370370001480000	ANES EPIDURAL 3 HR 30 MIN
370370001490000	ANES EPIDURAL 4 HR
370370001490001	ANES EPIDURAL SETUP
370370019960000	ANES DAILY MGMT CONT DRUG ADMIN EPID/SUBARACH
370370990120000	ANES EPIDURAL 1 MIN
960963019960000	PF ANES DAILY MGMT CONT DRUG ADMIN EPID/SUBARACH
970975622750000	PF INJ ANES EPIDURAL CERVICAL/THORACIC SINGLE
970975622780000	PF INJ ANES EPIDURAL LUMBAR/CAUDAL SINGLE
970975622790000	PF INJ ANES EPIDURAL LUMBAR/CAUDAL CONTINUOUS
970975622890000	PF INJ EPIDURAL LUMBAR/CAUDAL
970975622980000	PF INJ EPIDURAL CERVICAL/THORACIC
970975623550000	PF REMOVE INTRATHECAL/EPIDURAL CATHETER
980980623100000	PF INJ SINGLE EPID/SUBARACH CERV/THOR
980980623110000	PF INJ SINGLE EPID/SUBARACH LUMB/SACRAL
980980623180000	PF INJ W CATH PLACEMENT EPID/SUBARACH CERV/THOR
980980623190000	PF INJ W CATH PLACEMENT EPID/SUBARACH LUMB/SACRAL

Table S4. Standard Charge Codes for Liposomal Bupivacaine

Standard Charge Code	Standard Charge Description
110229000010000	PAYMENT/ADJUSTMENT
250250073230000	MISC ANALGESIC
250250111850000	BUPIVACAINE, EXPAREL VL 13.3MG/ML (1.3%) 10ML
250250111860000	BUPIVACAINE, EXPAREL VL 13.3MG/ML (1.3%) 20ML
250250120150000	BUPIVACAINE, EXPAREL INJ 1MG
250888002710000	BUPIVACAINE PARENTERAL MISC
250888026460000	BUPIVACAINE (EXPAREL) PARENTERAL MISC
250999002100000	BUPIVACAINE MISC
99999	UNKNOWN[a]

[a]Standard charge code corresponds to hospital charge identification 318917179, 338547263, and 355737239 and hospital charge description liposomal bupivacaine PF 1 mg (260)/20 mL.

Table S5. Complete Regression Tables for Economic and Clinical Outcomes

Parameter	Description	*df*	Estimate	Standard Error	Wald 95% Confidence Limits	Wald χ^2	*P* Value
**A. Total Hospital Cost**							
Intercept	—	1	10.4315	0.0551	10.3236, 10.5394	35 887.9	<.0001
Procedure	COLORECTAL	1	-0.1651	0.018	-0.2003, -0.1299	84.49	<.0001
Procedure	ABDOMEN	0	0	0	0, 0	—	—
Epidural	1	1	0.2078	0.0226	0.1634, 0.2521	84.46	<.0001
Epidural	0	0	0	0	0, 0	—	—
AGE	—	1	0.0028	0.0007	0.0014, 0.0041	16.18	<.0001
GENDER	F	1	-0.0724	0.016	-0.1038, -0.041	20.46	<.0001
GENDER	M	0	0	0	0, 0	—	—
Race	Other	1	-0.0511	0.0204	-0.091, -0.0112	6.31	.012
Race	White	0	0	0	0, 0	—	—
CCI	—	1	0.0413	0.0033	0.035, 0.0477	161.4	<.0001
Year	2016	1	0.0832	0.0234	0.0373, 0.1291	12.62	.0004
Year	2017	1	0.0059	0.0232	-0.0396, 0.0513	0.06	.8004
Year	2018	1	0.032	0.0243	-0.0156, 0.0795	1.74	.1876
Year	2019	0	0	0	0, 0	—	—
TEACHING	NO	1	-0.0808	0.021	-0.1219, -0.0398	14.87	.0001
TEACHING	YES	0	0	0	0, 0	—	—
Location	RURAL	1	-0.0255	0.0277	-0.0799, 0.0288	0.85	.3573
Location	URBAN	0	0	0	0, 0	—	—
PROV_REGION	MIDWEST	1	-0.5675	0.043	-0.6517, -0.4833	174.48	<.0001
PROV_REGION	NORTHEAST	1	-0.5339	0.0387	-0.6097, -0.4581	190.54	<.0001
PROV_REGION	SOUTH	1	-0.5909	0.0346	-0.6588, -0.523	291.19	<.0001
PROV_REGION	WEST	0	0	0	0, 0	—	—
BEDS_GRP	000-199	1	-0.1469	0.0279	-0.2016, -0.0922	27.68	<.0001
BEDS_GRP	200-299	1	0.1277	0.0276	0.0737, 0.1818	21.44	<.0001
BEDS_GRP	300-399	1	-0.041	0.0245	-0.0889, 0.0069	2.81	.0937
BEDS_GRP	400-499	1	0.0259	0.0388	-0.0502, 0.1019	0.45	.5047
BEDS_GRP	500+	0	0	0	0, 0	—	—

Table S5. Complete Regression Tables for Economic and Clinical Outcomes

Parameter	Description	*df*	Estimate	Standard Error	Wald 95% Confidence Limits	Wald χ^2	*P* Value
Payor	MEDICAID	1	0.2319	0.0322	0.1687, 0.2951	51.78	<.0001
Payor	MEDICARE	1	0.1199	0.0221	0.0766, 0.1633	29.4	<.0001
Payor	OTHERS	1	0.1401	0.0299	0.0816, 0.1986	22.03	<.0001
Payor	COMMERCIAL	0	0	0	0, 0	—	—
**B. Length of Stay**							
Intercept	—	1	1.7836	0.0642	1.6577, 1.9095	771.18	<.0001
Procedure	COLORECTAL	1	-0.2345	0.0202	-0.2741, -0.1948	134.34	<.0001
Procedure	ABDOMEN	0	0	0	0, 0	—	—
Epidural	1	1	0.2147	0.0255	0.1647, 0.2648	70.67	<.0001
Epidural	0	0	0	0	0, 0	—	—
AGE	—	1	0.003	0.0008	0.0015, 0.0046	14.95	.0001
GENDER	F	1	-0.0233	0.0184	-0.0593, 0.0128	1.6	.2056
GENDER	M	0	0	0	0, 0	—	—
Race	Others	1	-0.013	0.0233	-0.0587, 0.0327	0.31	.5776
Race	White	0	0	0	0, 0	—	—
CCI	—	1	0.0384	0.0036	0.0313, 0.0455	111.19	<.0001
Year	2016	1	0.0651	0.0267	0.0127, 0.1174	5.93	.0149
Year	2017	1	-0.034	0.0266	-0.086, 0.0181	1.63	.2014
Year	2018	1	-0.0382	0.0279	-0.0929, 0.0165	1.87	.1712
Year	2019	0	0	0	0, 0	—	—
TEACHING	NO	1	0.1623	0.024	0.1152, 0.2093	45.66	<.0001
TEACHING	YES	0	0	0	0, 0	—	—
Location	RURAL	1	-0.1469	0.0322	-0.2101, -0.0838	20.79	<.0001
Location	URBAN	0	0	0	0, 0	—	—
PROV_REGION	MIDWEST	1	-0.1004	0.05	-0.1983, -0.0024	4.04	.0446
PROV_REGION	NORTHEAST	1	-0.0536	0.045	-0.1418, 0.0345	1.42	.2328
PROV_REGION	SOUTH	1	-0.1114	0.0408	-0.1913, -0.0315	7.47	.0063
PROV_REGION	WEST	0	0	0	0, 0	—	—
BEDS_GRP	000-199	1	-0.2716	0.0322	-0.3347, -0.2085	71.18	<.0001
BEDS_GRP	200-299	1	-0.0445	0.0315	-0.1063, 0.0173	1.99	.1584

Table S5. Complete Regression Tables for Economic and Clinical Outcomes

Parameter	Description	*df*	Estimate	Standard Error	Wald 95% Confidence Limits	Wald χ^2	*P* Value
BEDS_GRP	300-399	1	-0.1142	0.028	-0.1692, -0.0592	16.57	<.0001
BEDS_GRP	400-499	1	-0.0577	0.0443	-0.1446, 0.0292	1.7	.1928
BEDS_GRP	500+	0	0	0	0, 0	—	—
Payor	MEDICAID	1	0.3391	0.0367	0.2671, 0.4111	85.16	<.0001
Payor	MEDICARE	1	0.1811	0.0254	0.1312, 0.2309	50.64	<.0001
Payor	OTHERS	1	0.1713	0.0343	0.104, 0.2386	24.89	<.0001
Payor	COMMERCIAL	0	0	0	0, 0	—	—
**C. Discharge to Home**							
Intercept	—	1	6.4335	0.3542	5.7392, 7.1278	329.86	<.0001
Procedure	COLORECTAL	1	0.7369	0.0854	0.5695, 0.9043	74.44	<.0001
Procedure	ABDOMEN	0	0	0	0, 0	—	—
Epidural	1	1	-0.672	0.1046	-0.8771, -0.4669	41.24	<.0001
Epidural	0	0	0	0	0, 0	—	—
AGE	—	1	-0.059	0.0044	-0.0677, -0.0504	178.02	<.0001
GENDER	F	1	0.0062	0.0814	-0.1534, 0.1658	0.01	.9394
GENDER	M	0	0	0	0, 0	—	—
Race	Others	1	-0.1464	0.1046	-0.3515, 0.0586	1.96	.1617
Race	White	0	0	0	0, 0	—	—
CCI	—	1	-0.1334	0.0141	-0.161, -0.1059	90.15	<.0001
Year	2016	1	-0.2004	0.1169	-0.4296, 0.0287	2.94	.0865
Year	2017	1	-0.024	0.1184	-0.2561, 0.2081	0.04	.8393
Year	2018	1	0.0788	0.1266	-0.1693, 0.3268	0.39	.5338
Year	2019	0	0	0	0, 0	—	—
TEACHING	NO	1	-0.408	0.1063	-0.6164, -0.1997	14.73	.0001
TEACHING	YES	0	0	0	0, 0	—	—
Location	RURAL	1	0.1103	0.1313	-0.1471, 0.3676	0.71	.401
Location	URBAN	0	0	0	0, 0	—	—
PROV_REGION	MIDWEST	1	-0.1933	0.2127	-0.6102, 0.2236	0.83	.3634
PROV_REGION	NORTHEAST	1	-0.2015	0.193	-0.5798, 0.1768	1.09	.2965
PROV_REGION	SOUTH	1	0.3305	0.1774	-0.0172, 0.6782	3.47	.0625
PROV_REGION	WEST	0	0	0	0, 0	—	—
BEDS_GRP	000-199	1	0.2269	0.1417	-0.0508, 0.5046	2.57	.1092
BEDS_GRP	200-299	1	-0.0726	0.1315	-0.3303, 0.1851	0.31	.5806

Table S5. Complete Regression Tables for Economic and Clinical Outcomes

Parameter	Description	*df*	Estimate	Standard Error	Wald 95% Confidence Limits	Wald χ^2	*P* Value
BEDS_GRP	300-399	1	0.0305	0.1228	-0.2101, 0.2711	0.06	.8038
BEDS_GRP	400-499	1	0.0495	0.1865	-0.316, 0.4149	0.07	.7908
BEDS_GRP	500+	0	0	0	0, 0	—	—
Payor	MEDICAID	1	-0.8874	0.2094	-1.2977, -0.4771	17.97	<.0001
Payor	MEDICARE	1	-0.9617	0.1359	-1.228, -0.6955	50.11	<.0001
Payor	OTHERS	1	-0.5907	0.2105	-1.0033, -0.1782	7.88	.005
Payor	COMMERCIAL	0	0	0	0, 0	—	—
**D. 30-Day Readmission**							
Intercept	—	1	-3.1331	0.3569	-3.8326, -2.4336	77.07	<.0001
Procedure	COLORECTAL	1	-0.1227	0.0994	-0.3175, 0.0721	1.52	.2171
Procedure	ABDOMEN	0	0	0	0, 0	—	—
Epidural	1	1	0.3226	0.1203	0.0868, 0.5583	7.19	.0073
Epidural	0	0	0	0	0, 0	—	—
AGE	—	1	-0.0009	0.004	-0.0087, 0.0069	0.05	.8223
GENDER	F	1	0.045	0.0896	-0.1306, 0.2206	0.25	.6153
GENDER	M	0	0	0	0, 0	—	—
Race	Others	1	0.1906	0.1092	-0.0235, 0.4047	3.04	.081
Race	White	0	0	0	0, 0	—	—
CCI	—	1	0.0616	0.0167	0.0288, 0.0943	13.59	.0002
Year	2016	1	0.242	0.1418	-0.0359, 0.5198	2.91	.0879
Year	2017	1	0.3014	0.139	0.0289, 0.5738	4.7	.0301
Year	2018	1	0.3325	0.1462	0.0459, 0.6191	5.17	.023
Year	2019	0	0	0	0, 0	—	—
TEACHING	NO	1	-0.079	0.1176	-0.3094, 0.1514	0.45	.5016
TEACHING	YES	0	0	0	0, 0	—	—
Location	RURAL	1	0.0309	0.1541	-0.2712, 0.333	0.04	.8412
Location	URBAN	0	0	0	0, 0	—	—
PROV_REGION	MIDWEST	1	0.4655	0.2875	-0.0979, 1.029	2.62	.1054
PROV_REGION	NORTHEAST	1	0.6404	0.2598	0.1313, 1.1496	6.08	.0137
PROV_REGION	SOUTH	1	0.6973	0.245	0.217, 1.1775	8.1	.0044
PROV_REGION	WEST	0	0	0	0, 0	—	—
BEDS_GRP	000-199	1	-0.1595	0.1698	-0.4922, 0.1732	0.88	.3474
BEDS_GRP	200-299	1	0.0688	0.1525	-0.2302, 0.3678	0.2	.652
BEDS_GRP	300-399	1	0.0031	0.1387	-0.2687, 0.275	0	.9822

Table S5. Complete Regression Tables for Economic and Clinical Outcomes

Parameter	Description	*df*	Estimate	Standard Error	Wald 95% Confidence Limits	Wald χ^2	*P* Value
BEDS_GRP	400-499	1	0.0527	0.217	-0.3727, 0.4781	0.06	.808
BEDS_GRP	500+	0	0	0	0, 0	—	—
Payor	MEDICAID	1	0.4697	0.1641	0.1481, 0.7914	8.19	.0042
Payor	MEDICARE	1	0.0951	0.1259	-0.1517, 0.3419	0.57	.4503
Payor	OTHERS	1	0.1111	0.1653	-0.213, 0.4351	0.45	.5017
Payor	COMMERCIAL	0	0	0	0, 0	—	—
**E. Hypotension**							
Intercept	—	1	-4.1445	0.3414	-4.8136, -3.4755	147.42	<.0001
Procedure	COLORECTAL	1	-0.4439	0.0943	-0.6287, -0.2592	22.17	<.0001
Procedure	ABDOMEN	0	0	0	0, 0	—	—
Epidural	1	1	1.0208	0.105	0.815, 1.2267	94.5	<.0001
Epidural	0	0	0	0	0, 0	—	—
AGE	—	1	0.0258	0.0043	0.0173, 0.0343	35.64	<.0001
GENDER	F	1	0.0337	0.0898	-0.1424, 0.2097	0.14	.7078
GENDER	M	0	0	0	0, 0	—	—
Race	Others	1	0.1341	0.1131	-0.0876, 0.3558	1.4	.2359
Race	White	0	0	0	0, 0	—	—
CCI	—	1	0.1001	0.0155	0.0698, 0.1304	41.96	<.0001
Year	2016	1	0.3146	0.1334	0.0531, 0.5761	5.56	.0184
Year	2017	1	0.1354	0.1368	-0.1327, 0.4034	0.98	.3223
Year	2018	1	0.3829	0.136	0.1164, 0.6494	7.93	.0049
Year	2019	0	0	0	0, 0	—	—
TEACHING	NO	1	-0.0432	0.1152	-0.269, 0.1825	0.14	.7073
TEACHING	YES	0	0	0	0, 0	—	—
Location	RURAL	1	-0.001	0.1469	-0.2889, 0.2869	0	.9945
Location	URBAN	0	0	0	0, 0	—	—
PROV_REGION	MIDWEST	1	0.0915	0.2215	-0.3427, 0.5257	0.17	.6795
PROV_REGION	NORTHEAST	1	-0.5763	0.2121	-0.9921, -0.1605	7.38	.0066
PROV_REGION	SOUTH	1	-0.1486	0.1841	-0.5093, 0.2121	0.65	.4194
PROV_REGION	WEST	0	0	0	0, 0	—	—
BEDS_GRP	000-199	1	0.0543	0.1602	-0.2597, 0.3682	0.11	.7349
BEDS_GRP	200-299	1	0.403	0.1424	0.1239, 0.6821	8.01	.0047
BEDS_GRP	300-399	1	-0.2949	0.1493	-0.5874, -0.0023	3.9	.0482
BEDS_GRP	400-499	1	-0.0353	0.2021	-0.4314, 0.3608	0.03	.8614

Table S5. Complete Regression Tables for Economic and Clinical Outcomes

Parameter	Description	*df*	Estimate	Standard Error	Wald 95% Confidence Limits	Wald χ^2	*P* Value
BEDS_GRP	500+	0	0	0	0, 0	—	—
Payor	MEDICAID	1	0.3757	0.1892	0.0048, 0.7466	3.94	.0471
Payor	MEDICARE	1	0.0779	0.1302	-0.1774, 0.3332	0.36	.5498
Payor	OTHERS	1	0.3749	0.1758	0.0304, 0.7194	4.55	.0329
Payor	COMMERCIAL	0	0	0	0, 0	—	—
**F. Vasopressor Use**							
Intercept	—	1	-4.5512	0.3998	-5.3348, -3.7675	129.57	<.0001
Procedure	COLORECTAL	1	-0.6646	0.1093	-0.8788, -0.4505	37	<.0001
Procedure	ABDOMEN	0	0	0	0, 0	—	—
Epidural	1	1	1.1386	0.1207	0.902, 1.3751	89.01	<.0001
Epidural	0	0	0	0	0, 0	—	—
AGE	—	1	0.0289	0.0051	0.0189, 0.0389	31.9	<.0001
GENDER	F	1	-0.3053	0.1063	-0.5137, -0.0969	8.24	.0041
GENDER	M	0	0	0	0, 0	—	—
Race	Others	1	0.2554	0.1306	-0.0006, 0.5113	3.82	.0505
Race	White	0	0	0	0, 0	—	—
CCI	—	1	0.0992	0.0181	0.0638, 0.1346	30.2	<.0001
Year	2016	1	0.3655	0.1583	0.0551, 0.6758	5.33	.021
Year	2017	1	0.2737	0.1611	-0.042, 0.5894	2.89	.0893
Year	2018	1	0.319	0.1641	-0.0027, 0.6407	3.78	.052
Year	2019	0	0	0	0, 0	—	—
TEACHING	NO	1	-0.0884	0.1365	-0.3559, 0.1791	0.42	.5172
TEACHING	YES	0	0	0	0, 0	—	—
Location	RURAL	1	0.0341	0.172	-0.303, 0.3713	0.04	.8428
Location	URBAN	0	0	0	0, 0	—	—
PROV_REGION	MIDWEST	1	0.2748	0.244	-0.2035, 0.753	1.27	.2601
PROV_REGION	NORTHEAST	1	-1.1789	0.259	-1.6865, -0.6713	20.72	<.0001
PROV_REGION	SOUTH	1	-0.2235	0.2047	-0.6247, 0.1777	1.19	.275
PROV_REGION	WEST	0	0	0	0, 0	—	—
BEDS_GRP	000-199	1	0.2829	0.1878	-0.0851, 0.6509	2.27	.1319
BEDS_GRP	200-299	1	0.5718	0.167	0.2445, 0.899	11.73	.0006
BEDS_GRP	300-399	1	-0.1527	0.1766	-0.4988, 0.1934	0.75	.3872
BEDS_GRP	400-499	1	-0.124	0.2353	-0.5852, 0.3372	0.28	.5983
BEDS_GRP	500+	0	0	0	0, 0	—	—

Table S5. Complete Regression Tables for Economic and Clinical Outcomes

Parameter	Description	*df*	Estimate	Standard Error	Wald 95% Confidence Limits	Wald χ^2	*P* Value
Payor	MEDICAID	1	0.4599	0.2226	0.0236, 0.8963	4.27	.0388
Payor	MEDICARE	1	0.1271	0.1556	-0.1778, 0.432	0.67	.4141
Payor	OTHERS	1	0.3562	0.2125	-0.0603, 0.7727	2.81	.0937
Payor	COMMERCIAL	0	0	0	0, 0	—	—

Abbreviation: *df*, degrees of freedom.
